# Ultrasonic‐assisted extraction of nanocellulose from sweet potato residue and its application in noodles

**DOI:** 10.1002/fsn3.4489

**Published:** 2024-09-25

**Authors:** Xueli Gao, Guanghui Li, Yonghui Wang, Weiyun Guo, Shenghua He, Jihong Huang

**Affiliations:** ^1^ Food and Pharmacy College, Xuchang University Xuchang China; ^2^ Collaborative Innovation Center of Functional Food by Green Manufacturing, Henan Province Xuchang China; ^3^ College of Agriculture, Henan University Zhengzhou China

**Keywords:** nanocellulose, noodles, sweet potato residue, ultrasonic‐assisted extraction

## Abstract

Enhancing the amount of dietary fiber without compromising the quality of noodles remains a challenge in the food industry. This study aimed to optimize the ultrasound‐assisted extraction parameters to enhance the yield of nanocellulose from sweet potato residue (SPR) using a response surface methodology. The impact of SPR nanocellulose on noodles' physicochemical properties was also explored. Results showed that the optimal extraction conditions for nanocellulose from SPR were identified as 180.73 U/mL cellulase concentration, 4.28 h ultrasonic time, 444.12 W ultrasonic power, and 14.17 h enzymatic hydrolysis time. The max yield of nanocellulose was 15.93% at optimum extraction conditions. Increasing the amount of SPR nanocellulose resulted in a reduction in the optimal cooking time, water absorption, elongation index, and springiness, as well as an increase in the breaking rate, cooking loss, hardness, gumminess, and chewiness of the noodles. Sensory analysis revealed higher acceptability of the noodles with 6%–12% SPR nanocellulose compared to other treatments. Microstructure demonstrated that noodles with 0%–18% SPR nanocellulose exhibited fewer holes and denser network structures, but higher SPR nanocellulose damaged the protein network. Those findings suggested that noodles with less than 12% SPR nanocellulose exhibited higher quality. This research provided the foundation for the development of nanocellulose‐enriched foods.

## Graphical abstract

Ultrasonic‐assisted extraction of nanocellulose from sweet potato residue. SPR nanocellulose improved noodles' physicochemical properties. Noodles with less than 12% SPR nanocellulose exhibited higher quality.

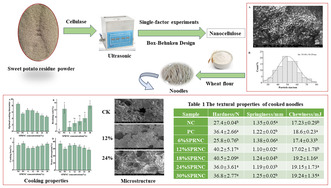

## 1 INTRODUCTION

Sweet potato residue (SPR) is the byproduct of the production of starch from fresh sweet potato, containing approximately 30% dietary fiber (DF), 4.1% protein, and 0.4% lipid at the dry weight (Dong et al., 2017). In China, the yearly production of sweet potatoes is 52 million tons, most of which are used in starch production. Production of 1 ton of starch usually generates about 4.5–5.0 tons of fresh SPR (Zhu et al., 2022). However, most SPRs are directly discarded, resulting in substantial environmental contamination and economic losses. Due to the nutritional values of SPR, numerous studies based on using SPR for component extraction (Arachchige et al., 2021; Lu et al., 2013; Mei et al., 2010) and food production (Kweman et al., 2021; Sun et al., 2023) have been reported.

Recently, nanocellulose (NC) has gained increasing interest from researchers and the food sector. NC usually has three types: cellulose nanocrystal, cellulose nanofibril, and bacterial cellulose (Zhang et al., 2021). NC derived from agro‐industrial by‐products (banana peel, straw, soybean root, etc.) has been extensively reported (Ebadi et al., 2021; Lu et al., 2021). Due to the advantages of NC (including large specific surface area, distinctive viscous characteristics, and excellent biocompatibility), it has been applied in the food industry as functional food ingredients, food additives, food packaging materials, and active substance carriers (Zhang et al., 2021). Qi et al. (2020) found that NC‐stabilized sausages exhibited higher fat and water‐binding capacities and stronger textural properties. Velásquez‐Cock et al. (2019) demonstrated the augmenting impact of cellulose nanofibrils on ice cream's mix viscosity, thereby influencing the fat destabilization without impacting ice crystal growth or the hardness of ice cream. Corral et al. (2017) reported that incorporating bacterial nanocellulose into wheat bread significantly increased specific volume and moisture retention while reducing bread crumbs' firmness and browning index.

Several methods have been employed for the preparation of NC from cellulosic materials. These methods include physical methods, such as ultrasound (Wu et al., 2023), grinding (Gemmer et al., 2022), microwave (Qu et al., 2022), and high‐pressure homogenization (Wu et al., 2021). Chemical treatments involved acid hydrolysis, alkali washing, and organic solvent methods (Jiang et al., 2023). Enzymatic hydrolysis approaches included endo‐xylanase, pectinase, manganese peroxidase, and so on. (Jiang et al., 2023). Typically, a combination of the two or more of the abovementioned methodologies has been adopted to produce NC. Guo et al. (2016) reported that the yield of cellulose nanocrystals increased from 18.3% to 52.8% using acid hydrolysis (45 min) during ultrasonic treatment. Zhou et al. (2018) found that ultrasound decreased the average length of the TEMPO‐NC from 292 to 185 nm. Squinca et al. (2020) demonstrated that the yield of NC from the ball‐milled cellulose pulp was 24.6% using 96 h of enzymatic hydrolysis and 5 min of sonication. Lim et al. (2024) noted that the highest yield of NC from raw durian husk fibers was 58.22% using the situ ultrasonication‐assisted one‐pot extraction method with a 7.5 min interval.

Noodles are one of China's beloved traditional staple foods, crafted from wheat flour with proportioned water and salt. With the increasing demand for healthy and safe food, various kinds of noodles, such as those enriched with mushrooms (Parvin et al., 2020), β‐glucan (Choo & Aziz, 2010), wheat bran (Song et al., 2013), and pumpkin and bean powder (Lee et al., 2002; Natocho et al., 2024), have been extensively investigated. Especially for noodles fortified with dietary fiber, low levels of dietary fiber can enhance the physicochemical characteristics of the noodles; nevertheless, excessive dietary fiber may lead to undesirable effects on the noodles' quality (Han et al., 2017). Therefore, increasing the amount of dietary fiber without affecting the quality of noodles poses a challenge for the food industry.

Therefore, the purpose of this study was to optimize ultrasound‐assisted extraction conditions to obtain the maximum yield of NC from SPR. Furthermore, the impact of NC from SPR (SPRNC) on the physicochemical properties of wheat noodles has also been investigated. The investigation provides a reference for the commercial manufacturing of noodles fortified with NC.

## 2 MATERIALS AND METHODS

### 2.1 Samples and reagents

SPR and dietary fiber extracted from sweet potato residue (SPRDF) were prepared in the lab of Jihong Huang at Xuchang University. The wheat flour, with a protein content ranging from 8.0% to 10.5%, was from Wudeli Flour Group (Daming, Hebei); the gluten (food grade, protein content 82.5%) and complex phosphate (food grade) were bought from Huafenfengye Co., Ltd. (Fengqiu, Henan); cellulase was obtained from Beijing Aoboxing Bio‐tech Co., Ltd. (Beijing, China); and glycerol was obtained from Haohua Chemical Reagent Co., Ltd. (Luoyang, Henan).

### 2.2 Preparation of SPRNC

With a slight modification, the method reported by Wu et al. (2023) was used to prepare NC. Briefly, 30 g of SPR powder was added to the glycerol solution (50%, 100 mL) and allowed to swell (3 h, 35°C). The mixture was subsequently centrifuged (2600 g, 5 min) and washed with water 2–3 times. The precipitate was the swollen SPR powder. Then, the precipitate and cellulase solution (100 mL) were mixed and hydrolyzed at 50°C. Subsequently, the mixtures were subjected to ultrasonic treatment at various times. The mixtures were centrifuged (2600 g, 15 min), then the supernatant was removed. Water was added to continue centrifugation until gelatinous appeared in the middle layer. The colloids were obtained and then dried at 80°C for 4 h. The SPRNC was stored at 4°C until used.

### 2.3 Determination of NC yield

The yield of nanocellulose extracted from SPR was calculated according to Equation (1):

(1) $$Y\left(\%\right)=\frac{\left(m_{2}-m_{1}\right)}{m_{3}}\times 100\%$$

Where *Y* (%) is the yield of NC; *m*_1_ (g) is the weight of the beaker after drying; *m*_2_ (g) is the total weight of the dried sample and the beaker; and *m*_3_ (g) is the weight of SPR powder.

### 2.4 Optimization of the extraction process of SPRNC

#### 2.4.1 Single‐factor experiments

The suitable values from the four variables, including cellulase concentration (50, 100, 150, 200, and 250 U/mL), ultrasonic time (3, 4, 5, 6, and 7 h), ultrasonic power (100, 200, 300, 400, and 500 W), and enzymatic hydrolysis time (1, 5, 10, 15, and 20 h), were identified and chosen using the single‐factor experimental design (SFED).

#### 2.4.2 Response surface methodology design

Based on the results obtained from SFED experiments, the optimization conditions for NC extracted from SPR were determined using a Box–Behnken Design (BBD) in the response surface methodology (RSM). The four independent variables considered in this study were cellulase concentration (X_1_), ultrasonic time (X_2_), ultrasonic power (X_3_), and enzymatic hydrolysis time (X_4_), and the dependent variable was the yield of NC (*Y*). Twenty‐nine experimental runs were carried out to optimize the extraction parameters. Three levels (−1, 0, +1) were used for each independent variable. Table 1 presents the independent variables with their levels and codes.

**TABLE 1:** Independent variables and levels used in Box–Behnken design (BBD).

Levels	Independent variables			
	X_1_: cellulase concentration (U/mL)	X_2_: ultrasonic time (h)	X_3_: ultrasonic power (W)	X_4_: enzymatic hydrolysis time (h)
−1	100	4	300	5
0	150	5	400	10
1	200	6	500	15

### 2.5 Preparation of SPRNC noodles

Based on the procedure described by Zhou et al. (2023) with some modifications, noodles were prepared as follows: a total of 100 g of blended flour, comprising 1.7% gluten, 0.3% complex phosphate, and SPRNC at varying concentrations of 6%, 12%, 18%, 24%, and 30%, were accurately weighed. Then, 33 g of distilled water was added to the flour and mixed until a smooth dough was formed. The doughs were rested in self‐sealing bags for 30 min at 25°C before passing through a noodle machine (ZH‐57, Baoding Tang'an E‐commerce Co., Ltd., China). The noodles were subsequently dried at ambient temperature until their moisture content fell below 12%, followed by sealing with plastic bags until use. Noodles without SPRNC and those with 6% SPRDF were used as the negative and positive controls.

### 2.6 Cooking properties

The optimal cooking time and cooking loss were assessed following the process of Xu et al. (2023) with minor modifications. Optimal cooking time: 30 noodles, each 10 cm in length, were selected and subsequently put in a beaker containing boiling water (500 mL). The optimal cooking time was the time when the white core of the noodles disappeared during the cooking process. Cooking loss: Noodles (15 g) were boiled in water (500 mL) for the optimal cooking time. After the noodles' preparation, the residual liquid was collected and subjected to evaporation on an electric stove until most of the water had dissipated. Finally, the remaining liquid was dried (105°C, 12 h). The cooking loss was determined as the ratio of the mass of dried matter in the soup to that of dried noodles.

According to Xiong et al. (2021), with some modifications, the water absorption was assessed as follows: 30 strips of noodles were precisely weighted and subsequently cooked in boiling water (500 mL) for the optimal cooking time. Water absorption was calculated as the ratio of the increased weight of cooked noodles to that of uncooked noodles.

The breaking rate was detected according to the approach presented by Shi et al. (2023). Thirty sticks of noodles with a length of 10 cm were immersed in the boiling water (500 mL), followed by cooking for optimal cooking time and draining. The breaking rate was determined as the ratio of the number of broken noodles to that of dried noodles.

The elongation index was assessed, followed by Vijayakumar et al. (2010) with slight modifications. Thirty noodles with a length of 10 cm were placed in 500 mL of boiling water and boiled for the optimal cooking time. Then, the noodles were taken out of the water, and the length of the cooked noodles was measured. The elongation index was the ratio of the increased length of cooked noodles to that of uncooked noodles.

### 2.7 Texture

The texture of the cooked noodles was determined using a texture analyzer (JY046, Beijing Yingsheng Hengtai Technology Co. Ltd.), based on the protocol described by Li et al. (2023). In brief, 30 strips of noodles with a length of 10 cm were put in boiling water (500 mL) and subsequently cooked for the optimal cooking time. Then, the cooked noodles were immersed in chilled water for 20 s. After draining, the cooked noodles were sealed with plastic bags until used. Three cooked noodles in parallel were compressed using the parameters: thick flat bottom probe 50 mm, compression ratio 60%, test speed 60 mm/min, induction force 0.5 N, and interval time 2 s.

### 2.8 Microstructure

According to Li et al. (2023), scanning electron microscopy (SEM; NovananoSEM450, FEI) was employed to observe the microstructure of the dried noodles' cross section and the SPRNC at a magnification of either 150,000 or 2000, with an operating voltage of either 10 or 5 kV.

### 2.9 Sensory evaluation

The sensory analysis was conducted by 20 food science major students (aged 18–24 years) at Xuchang University. All noodles were cooked for the optimal cooking time, and the coded samples were randomly assigned to the panelists. Before the experiment, the panelists were systemically trained. Using a nine‐point hedonic analysis, the panelists were instructed to score each sample based on noodles' appearance, mouthfeel, texture, color, and overall acceptability. The highest value indicated the highest degree of acceptance (9 = like extremely, 1 = dislike extremely).

### 2.10 Statistical analysis

Each test was carried out in triplicate, and the results were expressed as mean ± standard deviation. Statistical analysis was performed using Duncan's test by SPSS software (version 26, SPSS Inc., USA). A *p* < .05 was considered statistically significant. Moreover, the Box–Behnken design was analyzed by Design Expert 13.

## 3 RESULTS AND DISCUSSION

### 3.1 Single‐factor analysis of SPRNC extraction

**FIGURE 1:**
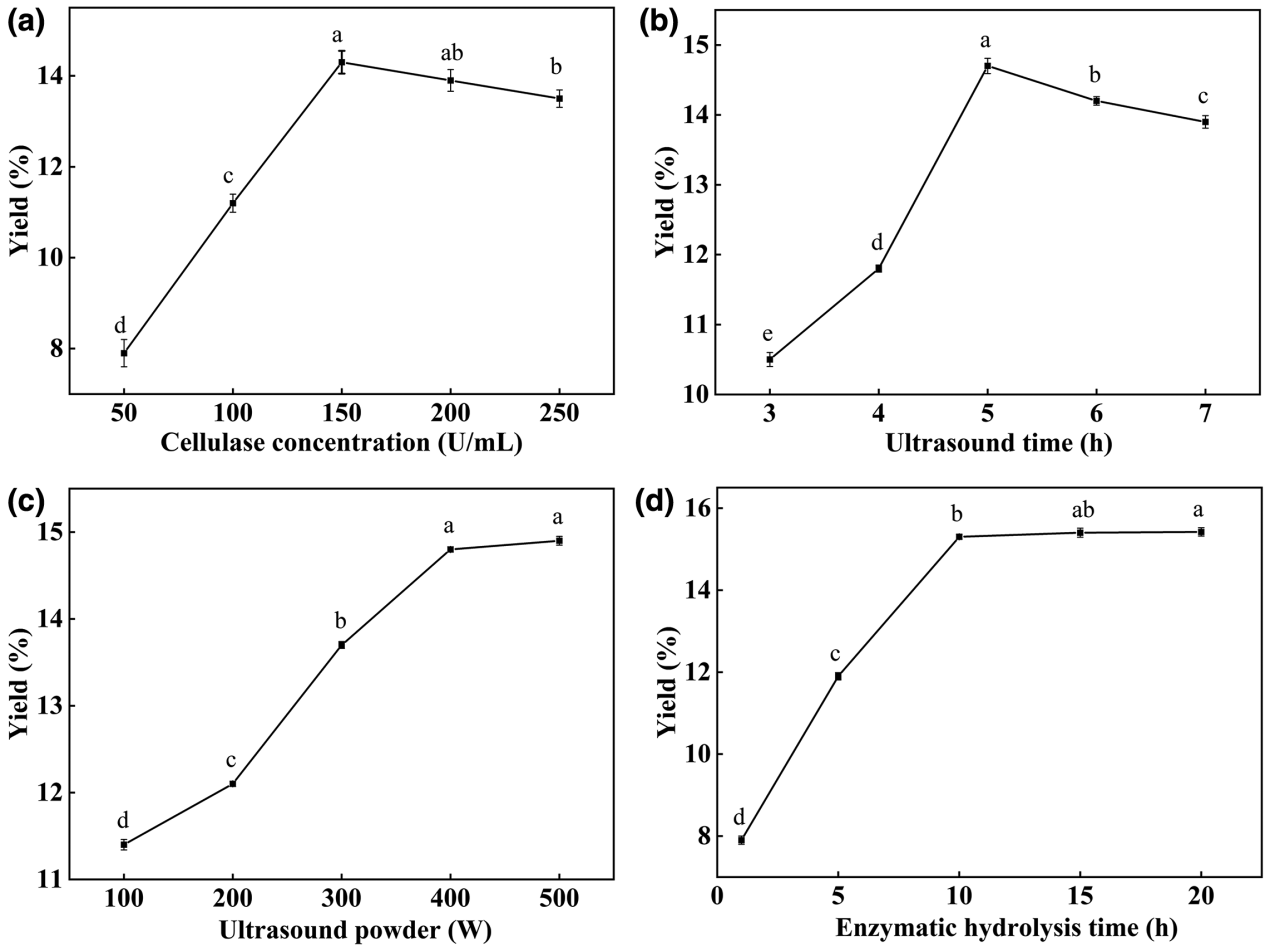
Effects of the cellulase concentration (a), ultrasonic time (b), ultrasonic power (c), and enzymatic hydrolysis time (d) on the yield of SPRNC. SPRNC, nanocellulose extracted from sweet potato residue.

The impact of cellulase concentration, ultrasonic time, ultrasonic power, and enzymatic hydrolysis time on the yield of SPRNC is shown in Figure 1. The yield of SPRNC exhibited a significant increase when the cellulase concentration was below 150 U/mL, followed by a gradual decline as the cellulase concentration exceeded 150 U/mL (Figure 1a). This may be due to the fact that excessive hydrolysis of SPR occurred when the cellulase concentration exceeded 150 U/mL. This result agreed with Wu et al. (2023), showing that the yield of NC initially increased and subsequently decreased with the ratios of pectinase and cellulase increased from 1:0 to 7:3. Therefore, cellulase concentrations ranging from 100 to 200 U/mL were chosen for the subsequent experiments.

The yield of SPRNC showed an increased trend with the prolongation of ultrasonic time, peaking at 5 h (Figure 1b). However, the yield decreased as the time increased from 5 to 7 h. More prolonged exposure may lead to NC degradation. This disagreed with Cui et al. (2016), who showed that nanocrystalline cellulose increased with the ultrasonic time increased from 30 to 60 min. Hence, the ultrasonic time between 4 and 6 h was chosen for the next experiment.

With an increase in ultrasonic power from 100 to 400 W, a significant enhancement was observed in the yield of SPRNC. However, when ultrasonic power was above 400 W, yields did not change significantly (Figure 1c). This may be due to the fact that ultrasonic with higher power made the cellulose into finer cellulose, and the specific surface area increased, which was beneficial for enzymatic hydrolysis. Abd Hamid et al. (2016) reported that as the ultrasonication power increased from 50 to 225 W, the yield of NC increased; however, no further increase in the yield of NC was obtained with the higher ultrasonication powder. Therefore, the ultrasound power was optimized between 300 and 500 W in the following experiments.

The enzymatic hydrolysis time considerably impacted the yield of NC from SPR (Figure 1d). The yield of SPRNC increased with the increasing enzymatic hydrolysis time. When the hydrolysis time was at 10 h, the highest yield was obtained for SPRNC. After that, the yield remained stable with increasing time. This may be because increasing the hydrolysis time damaged the crystalline regions and chemical bonds of SPR cellulose. However, a longer time would decrease the enzyme concentration, and the enzymatic reaction was completed. This was agreed with Cui et al. (2016), who demonstrated that the yield of nanocrystalline cellulose increased with the enzymolysis time increasing. Therefore, the enzymatic hydrolysis time between 5 and 15 h was chosen in the following experiments to obtain the highest yield of SPRNC.

### 3.2 RSM optimization of production conditions for SPRNC

#### 3.2.1 Response surface model analysis

The production conditions of SPRNC were optimized using the BBD experiment, and the results are presented in Table 2. The impact of cellulase concentration (X_1_), ultrasonic time (X_2_), ultrasonic power (X_3_), and enzymatic hydrolysis time (X_4_) on the yield of SPRNC (*Y*, %) can be represented by a second‐order polynomial equation, which is as follows:

**TABLE 2:** Box–Behnken design for preparation of SPRNC and results.

Runs	Coded variables				Uncoded variables				Yield (*Y*, %)
	X_1_	X_2_	X_3_	X_4_	X_1_: cellulase concentration (U/mL)	X_2_: ultrasonic time (h)	X_3_: ultrasonic power (W)	X_4_: enzymatic hydrolysis time (h)	
1	0	−1	0	1	150	4	400	15	15.1
2	−1	−1	0	0	100	4	400	10	13.7
3	0	1	1	0	150	6	500	10	14.4
4	−1	0	1	0	100	5	500	10	13.1
5	1	1	0	0	200	6	400	10	14.8
6	0	−1	1	0	150	4	500	10	14.1
7	0	−1	0	−1	150	4	400	5	13.6
8	0	1	0	1	150	6	400	15	15.3
9	1	−1	0	0	200	4	400	10	14.5
10	1	0	−1	0	200	5	300	10	13.6
11	0	0	0	0	150	5	400	10	15.1
12	−1	0	0	1	100	5	400	15	13.9
13	−1	1	0	0	100	6	400	10	14.3
14	0	1	−1	0	150	6	300	10	13.2
15	0	0	0	0	150	5	400	10	15.2
16	0	0	0	0	150	5	400	10	15.4
17	−1	0	0	−1	100	5	400	5	12.6
18	1	0	0	1	200	5	400	15	15.3
19	−1	0	−1	0	100	5	300	10	12.7
20	0	0	−1	−1	150	5	300	5	13.6
21	0	0	1	−1	150	5	500	5	13.4
22	0	0	−1	1	150	5	300	15	13.1
23	0	0	1	1	150	5	500	15	15.6
24	0	1	0	−1	150	6	400	5	14.2
25	1	0	1	0	200	5	500	10	15.4
26	1	0	0	−1	200	5	400	5	14.5
27	0	−1	‐1	0	150	4	300	10	13.7
28	0	0	0	0	150	5	400	10	15.1
29	0	0	0	0	150	5	400	10	15.3

Abbreviation: SPRNC, nanocellulose extracted from sweet potato residue.

*Y* (%) = −7.20750+ 0.06070X_1_+ 2.50833X_2_+ 0.04655X_3_–0.10866X_4_+ 0.00007X_1_X_3_+ 0.002X_2_X_3_+ 0.00135X_3_X_4_–0.00025X_1_^2^–0.31833X_2_^2^–0.000094X_3_^2^–0.01623X_4_^2^.

A significance test and analysis of variance (ANOVA) were used to assess the model's validity and predictability, with the corresponding results presented in Table 3. The statistical significance of the model was estimated using the *p*‐value and *F*‐value. The *p*‐value <.05 indicates a significant model, while the *p*‐value <.01 suggests an extremely significant model. The model that predicted the yield of SPRNC was significant (*p* < .001), and the lack of fit was insignificant (*p* = .0530) (Table 3). The model terms including X_1_, X_3_, X_4_, X_1_X_3_, X_3_X_4_, X_1_^2^, X_2_^2^, X_3_^2^, and X_4_^2^ exhibited statistical significance, and the terms X_2_ and X_2_X_3_ were insignificant. The correlation coefficient value (*R* ^2^) was determined to be 0.9421, suggesting that the regression model can explain 94.21% of the yield value. The value of the adjusted determination coefficient (*R*_Adj_^2^) was 0.9046. The results indicated that models can be used on behalf of the data within the experimental regions.

**TABLE 3:** ANOVA for the fitted models.

Source	Sum of squares	Df	Mean square	*F*‐value	*p*‐value	
Model	21.57	11	1.96	25.12	<.0001	Significant
X_1_‐cellulase concentration	5.07	1	5.07	64.94	<.0001	
X_2_‐ultrasonic time	0.1875	1	0.1875	2.4	.1396	
X_3_‐ultrasonic power	3.1	1	3.1	39.72	<.0001	
X_4_‐enzymatic hydrolysis time	3.41	1	3.41	43.72	<.0001	
X_1_X_3_	0.49	1	0.49	6.28	.0227	
X_2_X_3_	0.16	1	0.16	2.05	.1704	
X_3_X_4_	1.82	1	1.82	23.34	.0002	
X_1_^2^	2.58	1	2.58	33.06	<.0001	
X_2_^2^	0.6573	1	0.6573	8.42	.0099	
X_3_^2^	5.77	1	5.77	73.94	<.0001	
X_4_^2^	1.07	1	1.07	13.68	.0018	
Residual	1.33	17	0.0781			
Lack of fit	1.26	13	0.0969	5.7	.053	Not significant
Pure error	0.068	4	0.017			
Cor total	22.9	28				
*R* ^2^ = 0.9421		*R* ^2^_Adj_ = 0.9046		*R* ^2^_Pred_ = 0.8101		

#### 3.2.2 Analysis of the variable interaction

**FIGURE 2:**
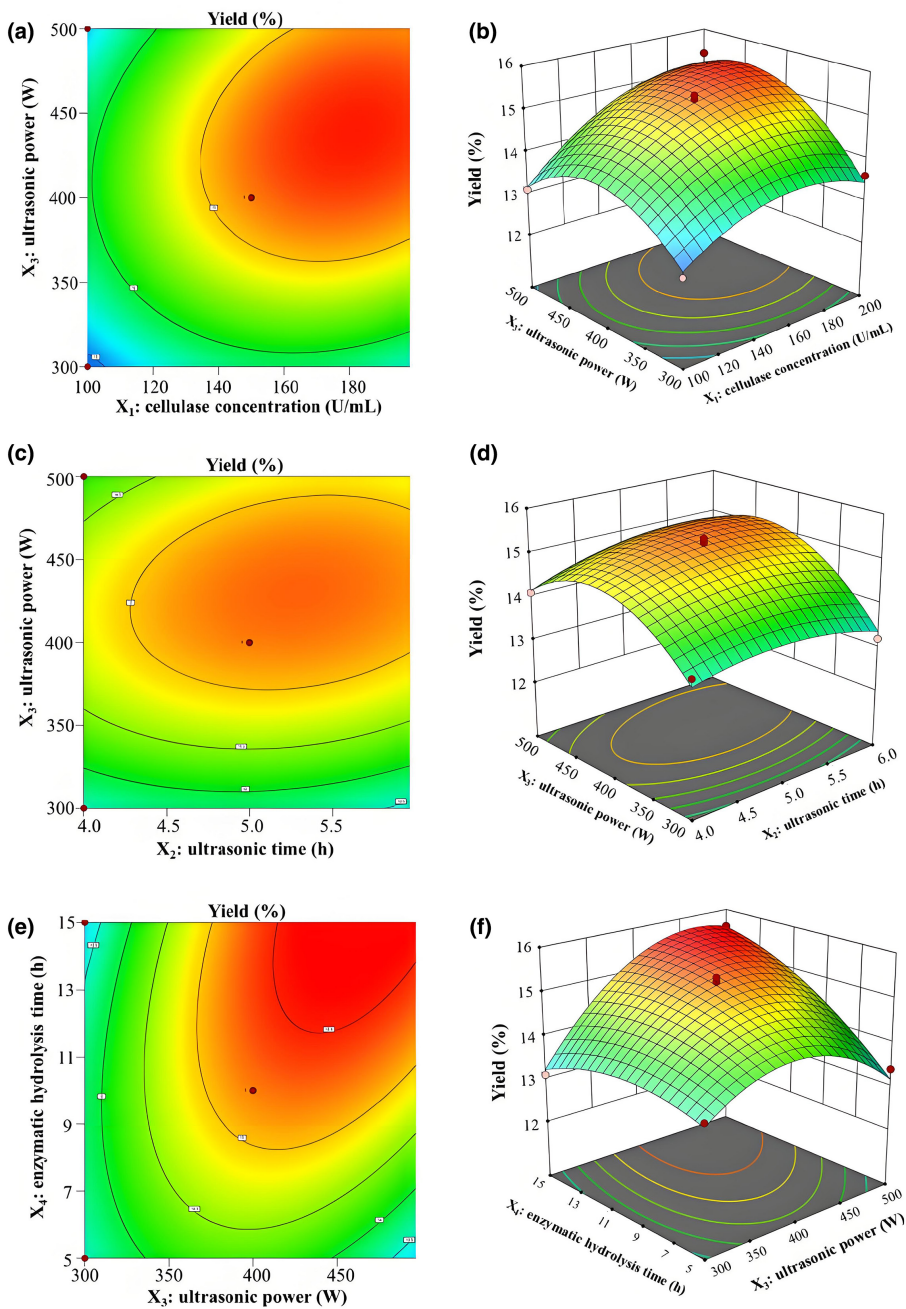
Contour plots (a, c, e) and response surface plots (b, d, f) of SPRNC affected by cellulase concentration (X_1_), ultrasonic time (X_2_), ultrasonic power (X_3_), and enzymatic hydrolysis time (X_4_). SPRNC, nanocellulose extracted from sweet potato residue.

Figure 2 displays the contour plots and three‐dimensional (3D) response surface, illustrating the intricate interplay among the pairwise factors. As shown in Figure 2a,b the yield decreased as the interaction between cellulase concentration and ultrasonic power increased until reaching an optimum point, beyond which it slowly decreased. Notably, the positive model term (+0.00007X_1_X_3_) indicated a synergistic effect that when both factors were reduced to their minimum values, there was a decrease in the yield of SPRNC.

Figure 2c,d represents the impact of the interaction between ultrasonic time and power on the yield of SPRNC. The positive model term (+0.002X_2_X_3_) suggested a synergistic effect between the ultrasonic time and power. It was noticed that the interaction between those two factors was found to be insignificant (*p* > .05, Table 3).

Figure 2e,f elucidates the response surface and contour plots depicting the interaction between ultrasonic power and enzymatic hydrolysis time. Compared to the interaction between cellulase concentration and ultrasonic power, the interaction between ultrasonic power and enzymatic hydrolysis time was more pronounced (Table 3). Interestingly, the positive model term (+0.00135X_3_X_4_) meant a synergistic effect between ultrasonic power and enzymatic hydrolysis time.

**FIGURE 3:**
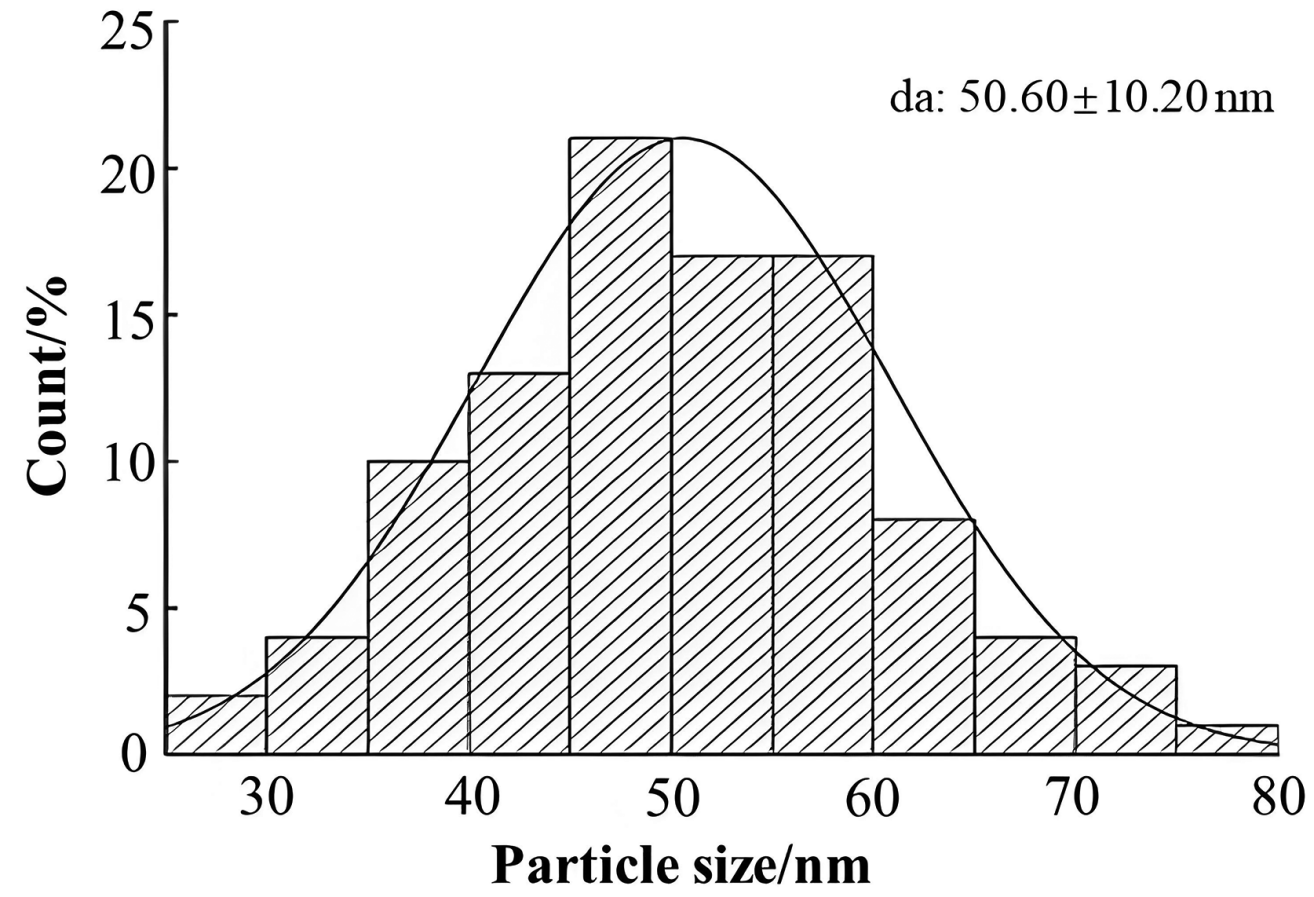
The particle size of SPRNC. SPRNC, Nanocellulose extracted from sweet potato residue.

#### 3.2.3 Validation studies

According to the results of Design Expert, the optimal conditions for the extraction of SPRNC were as follows: cellulase concentration of 180.73 U/mL, ultrasonic time of 4.28 h, ultrasonic power of 444.12 W, and enzymatic hydrolysis time of 14.17 h. The predicted yield value (*Y*) was found to be 15.61%. In the verification test conducted three times under these optimum conditions, the observed value was 15.93 ± 0.37%. This result confirmed the accuracy of the regression model used in this study. This was inconsistent with Squinca et al. (2020). Squinca et al. (2020) used a central composite design to optimize the extractive conditions for the cellulose nanocrystals, and the results showed that the yield (24.6%) of cellulose nanocrystals was obtained after 96 h of enzymatic hydrolysis of the ball‐milled cellulose pulp and 5 min of sonication. This difference may be caused by the raw material and the treatment time.

### 3.3 Characteristics of SPRNC

Figure 3 shows the particle size of SPRNC. SPRNC was an irregular sphere with relatively uniform size and distribution. Some particles exhibited agglomeration due to hydroxyl groups around the surface of SPRNC (Figure S1). This phenomenon can be attributed to the enzymatic hydrolysis by cellulase, which led to a reduction in cellulose size, an augmentation in the specific surface area, and enhanced hydrogen bonding among surface hydroxyl groups, resulting in agglomeration. As seen from Figure 3, the particle size of SPRNC ranged from 35 to 60 nm, and the average particle size was 50 nm. The results agreed with Chawla et al. (2023) and Lei et al. (2022). Chawla et al. (2023) reported that the particle size of NC derived from corn husks was 149.67 nm. Lei et al. (2022) found that the average width of bamboo shoots in NC was 56.37 nm, with a height of 7.44 nm.

### 3.4 Cooking properties

**FIGURE 4:**
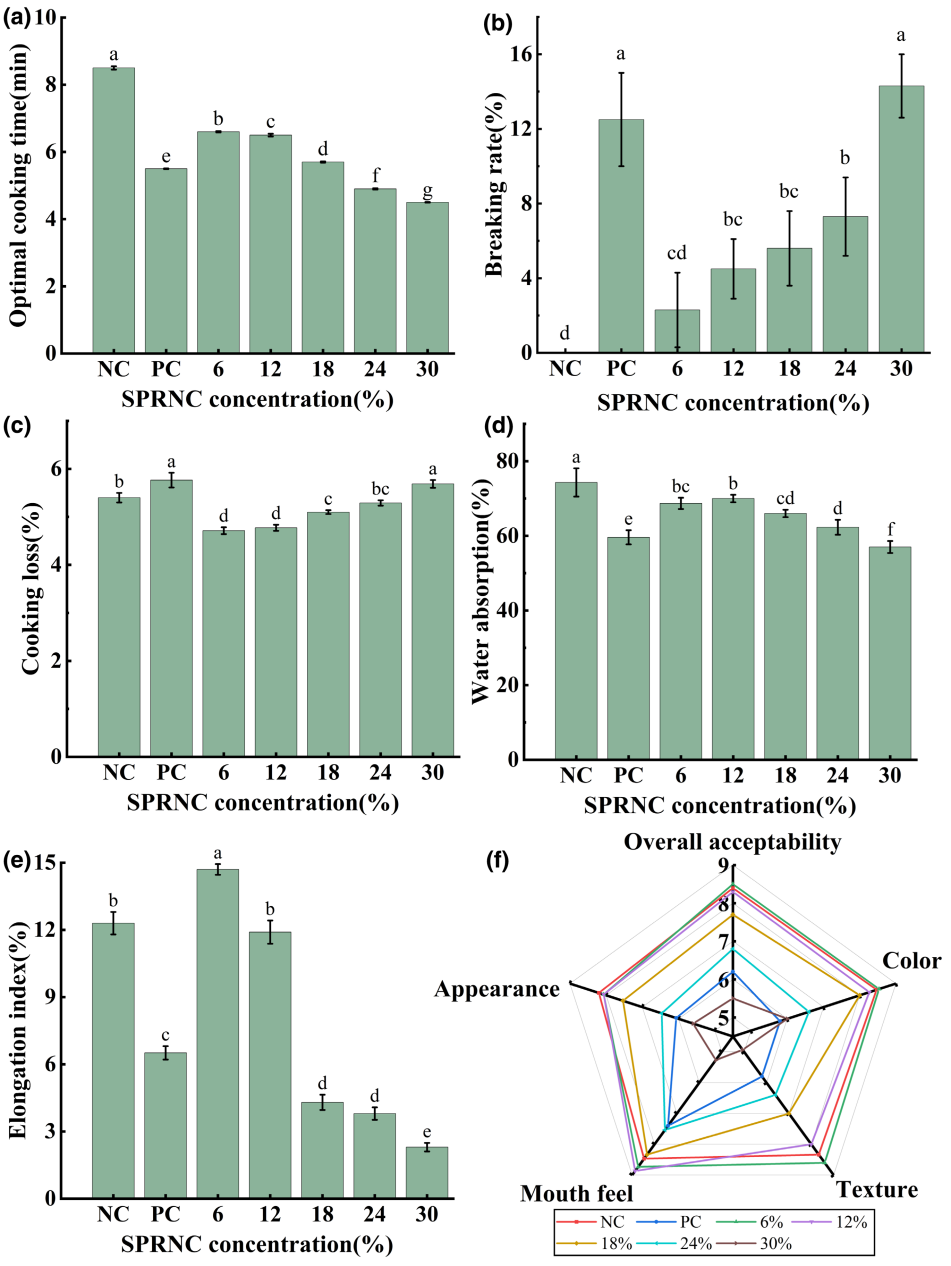
The cooking and sensory properties of noodles containing SPRNC. (a) The optimal cooking time; (b) breaking rate; (c) cooking loss; (d) water absorption; (e) the elongation index; (f) sensory properties. NC, the negative control; PC, the positive control; SPRNC, nanocellulose extracted from sweet potato residue.

Figure 4 shows the cooking properties of noodles containing SPRNC. The addition of SPRNC significantly reduced the optimal cooking time of the noodles (8.5–4.5 min) (Figure 4a). This may be due to the fact that SPRNC damaged the gluten network of the noodles, causing water to enter the noodles quickly. This was inconsistent with Ning et al. (2022), who showed that the optimal cooking time of dried noodles increased with increasing the concentration of Passion fruit mesocarp flour.

The breaking rate of noodles (0%–14.3%) increased as the amount of SPRNC increased from 6% to 30% (Figure 4b). This was attributed to the fact that the higher SPRNC reduced the noodles' toughness, and thereby the network structure of the dough was unstable, and the gluten protein was damaged during cooking, leading to an increased breaking rate. The result was in accordance with Liu et al. (2023), who found that highland barley flour with bigger sizes increased the breaking rate of noodles.

From Figure 4c, the cooking loss increased significantly by the SPRNC. It was because the increased SPRNC would compete with the protein for water, thus modifying the structure of the gluten protein. As a result, some starch and SPRNC could not be completely wrapped by the gluten network and would be leached out into the water. This agreed with Sun et al. (2023), showing that the cooking loss of noodles increased from 7.09% to 7.92% as the amount of thermally assisted potassium carbonate treatment sweet potato residue increased.

As seen in Figure 4d, the water absorption decreased as the SPRNC concentration increased from 6% to 30%. This was because the SPRNC made the noodles' structure looser, resulting in less water retention capacity. The result agreed with Ning et al. (2022), who showed that adding 3%–9% Passion fruit mesocarp flour decreased water absorption of noodles.

Compared with the negative control, the elongation index increased when the amount of SPRNC was 6%; then, the elongation index decreased when the amount of SPRNC increased from 12% to 30%. The reason may be that SPRNC had strong water absorption, and a low amount of SPRNC was conducive to the transformation of ‐SH‐ and ‐S‐S‐ in the dough, increasing the elongation of noodles. However, a higher SPRNC would reduce the amount of water bound to the gluten protein, resulting in an insufficient expansion of the gluten network structure and decreased elongation.

In addition, the cooking qualities (the optimal cooking time, cooking loss, breaking rate, elongation index, and water absorption) of noodles with 6% SPRDF were below those of the noodles with 18% SPRNC. Therefore, the NC improved the quality and increased the amount of cellulose in noodles.

### 3.5 Texture

The impact of SPRNC on the textural properties of cooked noodles has been presented in Table 4. With the SPRNC increased, the hardness (27.4–40.5 N), gumminess (12.7–15.5 N), and chewiness (17.23–19.24 mJ) increased, and springiness (1.35–1.10 mm) decreased. There were no statistically significant variations observed in the hardness, gumminess, springiness, and chewiness between noodles containing 6% SPRNC and the negative control. However, when the addition of SPRNC exceeded 6%, the cooked noodles exhibited an improvement of hardness and gumminess, accompanied by a decrease in springiness. This may be due to the fact that an excessive amount of SPRNC impeded the establishment of the gluten network, thereby impacting the gluten's elasticity and extensibility while concurrently reducing air chambers in the noodles. Lei et al. (2021) demonstrated that the insoluble dietary fiber extracted from the wheat bran decreased the noodles' hardness and springiness. Zhang et al. (2019) found that noodles with low concentrations (4%) of wheat bran insoluble dietary fiber exhibited a decrease in springiness and an increase in hardness and chewiness. Huh et al. (2019) reported that the hardness of the soy noodle increased from 0.54 to 0.77 N with the amount of hydroxypropyl methylcellulose increased from 0% to 1.5%.

**TABLE 4:** The effects of SPRNC on the textural properties of cooked noodles*.

Sample	Hardness/*N*	Springiness/mm	Gumminess/*N*	Chewiness/mJ
NC	27.4 ± 0.04^b^	1.35 ± 0.05^a^	12.7 ± 0.72^b^	17.23 ± 0.29^b^
PC	36.4 ± 2.66^a^	1.22 ± 0.02^b^	13.6 ± 1.22^ab^	18.6 ± 0.23^a^
6%SPRNC	25.8 ± 0.76^b^	1.38 ± 0.06^a^	12.6 ± 0.53^b^	17.4 ± 0.33^b^
12%SPRNC	40.2 ± 5.17^a^	1.10 ± 0.02^c^	15.5 ± 1.76^a^	17.02 ± 1.78^b^
18%SPRNC	40.5 ± 2.09^a^	1.24 ± 0.04^b^	15.4 ± 0.56^a^	19.2 ± 1.16^a^
24%SPRNC	36.0 ± 3.61^a^	1.19 ± 0.03^b^	14.6 ± 1.30^ab^	19.15 ± 1.73^a^
30%SPRNC	36.8 ± 2.77^a^	1.25 ± 0.02^b^	15.3 ± 1.08^a^	19.24 ± 1.35^a^

*Results are expressed as mean ± standard deviation. Different lower‐case letters at the same column indicate a significant difference (*p* < 0.05). Abbreviations: NC, the negative control; PC, the positive control; SPRNC, nanocellulose extracted from sweet potato residue.

### 3.6 Sensory evaluation

The sensory properties of the noodles containing SPRNC are summarized in Figure 4f. All the subjective characteristics, including appearance, color, texture, and mouthfeel, were declined by the SPRNC. Compared to the negative control, noodles with 6% and 12% SPRNC showed no significant difference (*p* > .05) in all the sensory attributes. Noodles with 6% SPRNC had the highest overall acceptability, followed by those with 12% SPRNC and the negative control, while the further addition of SPRNC resulted in a notable decline in the overall acceptability of the noodles. Overall, noodles with 6%–12% SPRNC addition exhibited acceptable sensory properties. These results were consistent with the cooking and texture studies. Biscuits fortified with nanocellulose isolated from waste chili stems reported by Ma et al. (2023) showed similar results of sensory properties.

### 3.7 Microstructure

**FIGURE 5:**
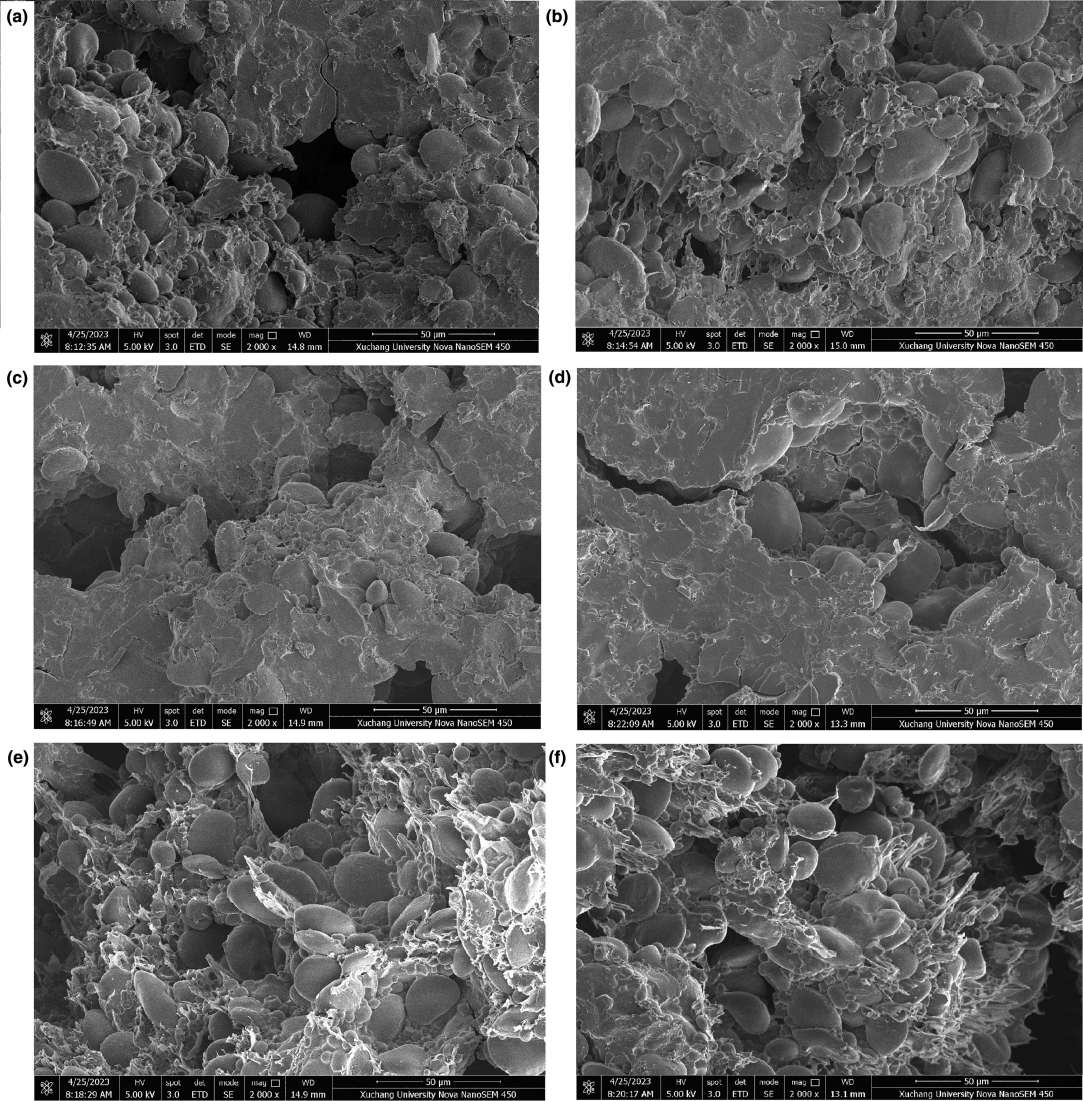
The microstructure of dried noodles with SPRNC. (a) Noodles without SPRNC; (b) noodles with 6% SPRNC; (c) noodles with 12% SPRNC; (d) noodles with 18% SPRNC; (e) noodles with 24% SPRNC; (f) noodles with 30% SPRNC. SPRNC, nanocellulose extracted from sweet potato residue.

Figure 5 displays the microstructure of noodles with SPRNC. Noodles with 0%–18% SPRNC exhibited fewer holes, and starch granules were consistently and evenly incorporated within the protein matrix, indicating the formation of a denser network structure (Figure 5a–e). However, when the SPRNC addition was high (24%–30%), the integrity of the protein network structure was compromised, resulting in inadequate embedding of starch granules in the protein matrix, and larger holes appeared. This may be due to the fact that low addition of SPRNC may fill gaps in the protein‐starch matrix, tightening the noodle structure. However, excessive SPRNC hindered the establishment of the protein network and weakened its strength after absorbing more water, leading to the separation of starch granules from the network. This phenomenon corresponded to the results of cooking loss and broken rate. Our results agreed with the microstructure of okara‐wheat noodles (Xie et al., 2023), and fermented semi‐dried rice noodles (Xiao et al., 2023). Xie et al. (2023) demonstrated that large holes and cracks appeared in the raw noodles as the okara addition increased from 15% to 25%. Xiao et al. (2023) reported that the starch‐protein network structure was damaged, and starch granules were not properly embedded in the protein network with the amount of sodium bicarbonate increased from 0.1% to 0.5%.

## 4 CONCLUSION

The NC was extracted from SPR using the ultrasonic‐assisted method in this study. The response surface methodology was applied to optimize the extraction conditions (cellulase concentration, ultrasonic time, ultrasonic power, and enzymatic hydrolysis time). Results indicated that NC yield was significantly influenced by ultrasonic power, cellulase concentration, and enzymatic hydrolysis time. The optimal extraction parameters were as follows: 180.73 U/mL cellulase concentration, 4.28 h ultrasonic time, 444.12 W ultrasonic power, and 14.17 h enzymatic hydrolysis time. Under these optimized conditions, the expected NC yield reached 15.61%. Furthermore, the impact of SPRNC on the cooking, texture, sensory quality, and microstructure of the noodles was also investigated. It has been confirmed that 6%–12% SPRNC had little effect on the noodles' quality, while higher SPRNC deteriorated the noodles' quality and damaged the protein network. Taken together, the amount of SPRNC in the noodles should not exceed 12%. Future research will focus on improving the quality of noodles with a high concentration of NC.

## AUTHOR CONTRIBUTIONS

**Xueli Gao:** Methodology (equal); validation (equal); writing – original draft (equal); writing – review and editing (equal). **Guanghui Li:** Supervision (equal); writing – original draft (equal); writing – review and editing (equal). **Yonghui Wang:** Validation (equal). **Weiyun Guo:** Funding acquisition (equal); project administration (equal); software (equal). **Shenghua He:** Writing – review and editing (equal). **Jihong Huang:** Supervision (equal); validation (equal).

## CONFLICT OF INTEREST STATEMENT

The authors declare that they have no known competing financial interests or personal relationships that could have appeared to influence the work reported in this paper.

## Supporting information


Figure S1.

## Data Availability

The data that support the findings of this study are available on request from the corresponding author.
