# Supplementary material for: Ultrasonic‐assisted extraction of nanocellulose from sweet potato residue and its application in noodles
Source: Food Sci Nutr. 2024 Sep 25;12(11):9175–86. doi: 10.1002/fsn3.4489 (PMC11606810; doi:10.1002/fsn3.4489)
Supplement: Supplementary file 1 — Figure S1. [file FSN3-12-9175-s001.pdf]

A

B

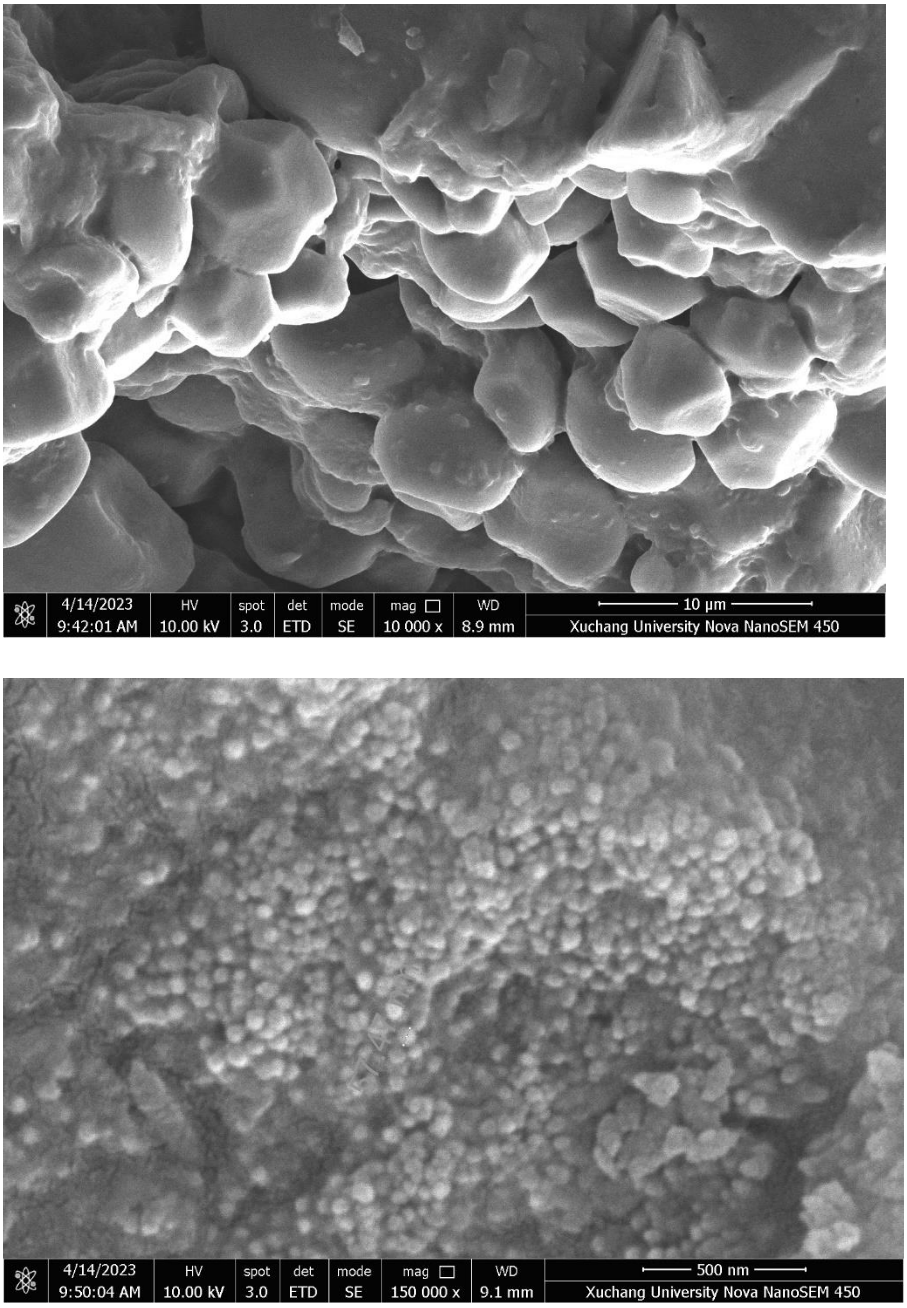

Figure S1 The microstructure of SPRNC (A:10000×, B: 150000×). SPRNC: nanocellulose extracted from sweet potato residue.
